# Safety, tolerability and effectiveness of HIV non-occupational prophylaxis in Taiwan

**DOI:** 10.7448/IAS.17.4.19736

**Published:** 2014-11-02

**Authors:** Hsing-Chuan Li, Yu-Ping Cheng, Chia-Jui Yang

**Affiliations:** 1 Department of Infection Control, Far Eastern Memorial Hospital, New Taipei City, Taiwan; 2 Department of Internal Medicine, Far Eastern Memorial Hospital, New Taipei City, Taiwan

## Abstract

**Introduction:**

Increasing numbers of new HIV infection is an important issue of public health in Taiwan. We aim to evaluate the safety, tolerability and effectiveness of HIV non-occupational prophylaxis (nPEP) in Taiwan.

**Materials and Methods:**

We conducted a prospective cohort observational study between March 2011 and May 2014. Three-combined antiretroviral agents were prescribed for all the persons who sought for HIV post-exposure prophylaxis after high risk sexual behaviour. HIV screening, health education and consultation were done before initiation of nPEP. Adverse effects were evaluated at Weeks 1, 2 and 4 and effectiveness was evaluated at Weeks 12 and 24. We also assessed adherence by pill count and regimen completion rates.

**Results:**

During the study periods, 255 persons were enrolled. Among the enrolled cases, 43.9% (112/255) of them received zidovudine (AZT)-based regimen while the others received tenofovir (TDF)-based regimen and the third agent was composed of mostly lopinavir/ritonavir (81.4%). The completion rate of nPEP was 85.9% (219/255), and discontinuation rate of nPEP among AZT-based regimen is higher than TDF-based regimen (17.0% vs 8.2%). Any grade adverse effects are higher among AZT-based regimen than TDF-based regimen (62.5% vs 32.1%) although most adverse effects were grade 1–2. After a 24-week follow-up, only one person experienced HIV seroconversion and he had primary syphilis at the moment when he sought for nPEP.

**Conclusions:**

NPEP could be an effective prevention method in a part of HIV prevention strategy and TDF-based regimen had better tolerability in Taiwan.

